# Explaining, not just predicting, drives interest in personal genomics

**DOI:** 10.1186/s13073-015-0188-5

**Published:** 2015-08-01

**Authors:** Susanne F. Meisel, Deanna Alexis Carere, Jane Wardle, Sarah S. Kalia, Tanya A. Moreno, Joanna L. Mountain, J. Scott Roberts, Robert C. Green, for the PGen Study Group

**Affiliations:** Cancer Research UK Health Behaviour Research Centre, Department of Epidemiology and Public Health, University College London, London, UK; Division of Genetics, Department of Medicine, Brigham and Women’s Hospital and Harvard Medical School, 75 Francis St, Boston, MA 02115 USA; Program in Genetic Epidemiology and Statistical Genetics, Department of Epidemiology, Harvard T.H. Chan School of Public Health, Boston, MA 02115 USA; Icahn Institute for Genomics and Multiscale Biology, Icahn School of Medicine at Mount Sinai, New York, NY 10029 USA; Millennium Health, San Diego, CA 92127 USA; 23andMe, Inc., 899 West Evelyn Avenue, Mountain View, CA 94041 USA; Department of Health Behavior & Health Education, University of Michigan School of Public Health, Ann Arbor, MI 48109 USA; Harvard Medical School, Boston, MA 02115 USA; Partners Personalized Medicine, EC Alumnae Building, Suite 301, 41 Avenue Louis Pasteur, Boston, MA 02115 USA

## Abstract

**Background:**

There is a widespread assumption that risk prediction is the major driver of customer interest in personal genomic testing (PGT). However, some customers may also be motivated by finding out whether their existing diseases have a genetic etiology. We evaluated the impact of an existing medical diagnosis on customer interest in condition-specific results from PGT.

**Methods:**

Using a prospective online survey of PGT customers, we measured customer interest prior to receiving PGT results for 11 health conditions, and examined the association between interest and personal medical history of these conditions using logistic regression.

**Results:**

We analyzed data from 1,538 PGT customers, mean age 48.7 years, 61 % women, 90 % White, and 47 % college educated. The proportion of customers who were ‘very interested’ in condition-specific PGT varied considerably, from 28 % for ulcerative colitis to 68% for heart disease. After adjusting for demographic and personal characteristics including family history, having a diagnosis of the condition itself was significantly associated with interest in genetic testing for risk of that condition, with odds ratios ranging from 2.07 (95 % CI 1.28-3.37) for diabetes to 19.99 (95 % CI 4.57-87.35) for multiple sclerosis.

**Conclusions:**

PGT customers are particularly interested in genetic markers for their existing medical conditions, suggesting that the value of genetic testing is not only predictive, but also explanatory.

**Electronic supplementary material:**

The online version of this article (doi:10.1186/s13073-015-0188-5) contains supplementary material, which is available to authorized users.

## Background

Direct-to-consumer (DTC) personal genomic testing (PGT) continues to be controversial, with debate centering on the analytical validity of PGT results, and the clinical validity and utility of returning this type of information to individual customers without involvement of healthcare providers [1–5]. Critics of PGT have cautioned that customers may misinterpret their genetic test results, leading to harm or unnecessary medical expenditure [1, 6]. However, other commentators have emphasized the potential for PGT to raise awareness of genetic disease predisposition and prompt lifestyle changes to mitigate disease risk; ultimately reducing the burden posed by these conditions on the healthcare system [7, 8].

Previous empirical research on the PGT consumer experience has focused primarily on the psychological and behavioral effects of PGT [9, 10]. Somewhat less attention has been paid to the motivations of individuals who seek PGT. In the few studies that have investigated this topic, the predominant drivers of interest in PGT have been identified as curiosity and gaining insight into future disease risk [10–12]. However, alternative motivations may also exist: for example, in two qualitative studies, we found that overweight and obese individuals sought genetic testing in part to obtain an explanation for their condition [13, 14]. As these findings were in a small sample, focused on only one health condition (obesity) and did not specifically pertain to DTC testing, it is not clear whether they could have application to the wider PGT consumer population.

In the traditional clinic setting, genetic testing related to a current diagnosis is frequently ordered for the purpose of identifying or confirming etiology, guiding treatment, determining prognosis, or providing recurrence risk information to families. To date, however, no quantitative study has investigated the effect of an existing medical diagnosis on interest in obtaining disease-specific genetic risk information in the DTC PGT setting. Using data from the Impact of Personal Genomics (PGen) Study, [15] a longitudinal survey study of customers from 23andMe, Inc. [16] and Pathway Genomics, [17] we evaluated the association between personal history of a disease, family history of a disease, and interest in PGT results for that disease. Defining personal and demographic correlates of interest in obtaining PGT is important because these could influence subsequent affective and behavioral responses to test results [10, 12]. Based on previous findings related to interest in genetic testing for obesity risk [13, 14], we hypothesized that PGT customers would be particularly interested in receiving genetic risk information for a health condition they already had, possibly even more than for a condition they might develop in the future.

## Materials and methods

### Study design

Data for our analysis were obtained from the Impact of Personal Genomics (PGen) Study, a longitudinal study of DTC PGT customers from 23andMe and Pathway Genomics. The PGen Study protocol was designed and administered by academic researchers at Harvard Medical School/Brigham and Women’s Hospital and the University of Michigan School of Public Health, in partnership with scientists from the two PGT companies, and independent survey design experts from SoundRocket (formerly Survey Sciences Group, Ann Arbor, MI, USA). Details of this academia-industry collaboration, and the analytical design and administration of the PGen Study, have been reported previously [15, 18].

Briefly, new DTC PGT customers of 23andMe and Pathway Genomics were recruited via email between March and July 2012. Survey data were collected online at three time points: first after customers purchased PGT, but before they received their results (baseline; BL), then approximately 2 weeks post results (2W), and finally approximately 6 months post results (6M). A total of 1,648 participants completed the BL survey. PGT results were returned to customers as per standard company practice, and then linked to survey data at the end of survey administration. The PGen Study was approved by the Partners Human Research Committee and the University of Michigan Health Sciences and Behavioral Sciences Institutional Review Board, and informed consent was obtained electronically from each participant prior to enrollment. The research conformed to the Helsinki Declaration.

### Outcome variables

For each condition addressed in the results report, customers of 23andMe and Pathway were provided with a personal risk estimate based on one or more single nucleotide polymorphism (SNP) markers. Interest in learning their risk estimate for each of 24 conditions was evaluated at BL with the multiple-choice question: ‘How interested are you in learning about your genetic risk for each of these diseases’, for which response options were: ‘not at all interested/somewhat interested’, and ‘very interested’.

Whether or not a customer had ever had a diagnosis of each condition for which a risk estimate could be obtained from PGT was evaluated at BL with the question: ‘Has a doctor ever told you that you have one of the following medical conditions’. Using a hierarchical question structure, participants were first presented with eight broad disease categories (neurological, psychiatric, gastrointestinal, heart, eye, diabetes, obesity, cancer), and then a choice of specific conditions for any selected category (for example, diabetes: Type 1 diabetes; Type 2 diabetes). They could select as many conditions as applicable.

Family history for each of the 24 conditions was evaluated at BL with the question: ‘Have any of your blood relatives (a parent, brother or sister, child, grandparent, aunt, uncle, or first cousin) ever had any of the following conditions’. The same hierarchical question structure (broad then specific) was used to identify family history.

The following demographic characteristics were recorded at BL: age, gender, race/ethnicity (six race categories, plus Hispanic/Latino ethnicity option), and highest education level (11 categories). Current health status was assessed at BL with one multiple-choice question from the Control Preference Scale [19]: ‘In general, would you say your health is: [excellent/very good/good/fair/poor]’. State anxiety, which may affect health information seeking, [20] was evaluated at BL with the two-item brief Generalized Anxiety Disorder screen (GAD-2) [21]. Multiple-choice responses to the two questions (‘Over the past two weeks, how often have you felt nervous, anxious, or on edge’ and ‘Over the past two weeks, how often have you been unable to stop or control worrying’) used response options from ‘not at all’ (scored 0) to ‘nearly every day’ (scored 3). A summed score of 4 or more represents a positive screen for generalized anxiety or panic disorder.

### Statistical analyses

Our analysis used survey data from only the BL survey, which comprised 240 questions, including demographic characteristics, interest, and motivations for undergoing PGT, and personal and family history. For the purposes of our analysis, we restricted the PGen Study dataset to the 1,648 participants who completed the BL survey prior to viewing their results. We further excluded participants with missing data on the predictor variables. Descriptive statistics were computed to summarize the baseline demographic characteristics of the study sample, interest in PGT results for each condition, and the frequency of personal and family history of each condition. Descriptive statistics were also computed within each company-specific sample to investigate differences in characteristics between participants recruited from each PGT company.

Of the 24 conditions for which customers of both companies were provided with PGT results, 13 conditions (age-related macular degeneration, Alzheimer’s disease, amyotrophic lateral sclerosis, blood clotting, breast cancer, celiac disease, chronic kidney disease, colorectal cancer, glaucoma, leukemia, lung cancer, Parkinson’s disease, and prostate cancer) had a reported frequency of <2%. These conditions were excluded from analysis because of the small sample sizes. Results for the following 11 conditions are reported here: asthma, bipolar disorder, type 2 diabetes, heart disease (specifically coronary artery disease), high cholesterol, multiple sclerosis, obesity, osteoarthritis, rheumatoid arthritis, skin cancer, and ulcerative colitis.

We used multivariate logistic regression to evaluate associations between personal history of a condition, family history of a condition, and interest in condition-specific PGT results. In all models, we dichotomized the outcome as ‘very interested’ vs. ‘somewhat/not at all interested’. We used binary variables for ‘ever’ personal or family history of each condition, and models were adjusted for variables likely to be associated with interest based on prior work [22, 23], including age (continuous), gender (male vs. female), race (White vs. non-White), Hispanic/Latino ethnicity (yes vs. no), education (four categories, as described in Table 1), positive GAD-2 screen for anxiety/panic disorder (yes vs. no), ever history of any other condition (yes vs. no), and current health status (five categories, as reported). Finally, all analyses were adjusted for PGT company (23andMe vs. Pathway) to account for the different recruitment strategies employed by each company and the resulting differences in demographic and motivational characteristics within the company-specific sub-groups. Data were analyzed using the Statistical Package for Social Sciences, SPSS version 21.0 (Chicago, IL, USA).

**Table 1:** Participant characteristics (n = 1,538)

Variable	Frequency
	n (%)
Mean age (SD; range)	48.7 (15.5; 20–95)
Female	936 (60.9)
Non-White race	154 (10.0)
Hispanic/Latino ethnicity	84 (5.5)
23andMe customers	978 (63.6)
Positive GAD-2 screen for anxiety/panic disorder	163 (10.6)
Highest level of education	
< College degree	340 (22.1)
College degree	472 (30.7)
Some graduate school	531 (34.5)
Doctoral degree	295 (12.7)
Self-reported health	
Poor	65 (4.2)
Fair	172 (11.2)
Good	465 (30.3)
Very good	604 (39.3)
Excellent	231 (15.0)

GAD-2, Generalized Anxiety Disorder – 2 Item Scale; SD, standard deviation

## Results

**Fig. 1:**
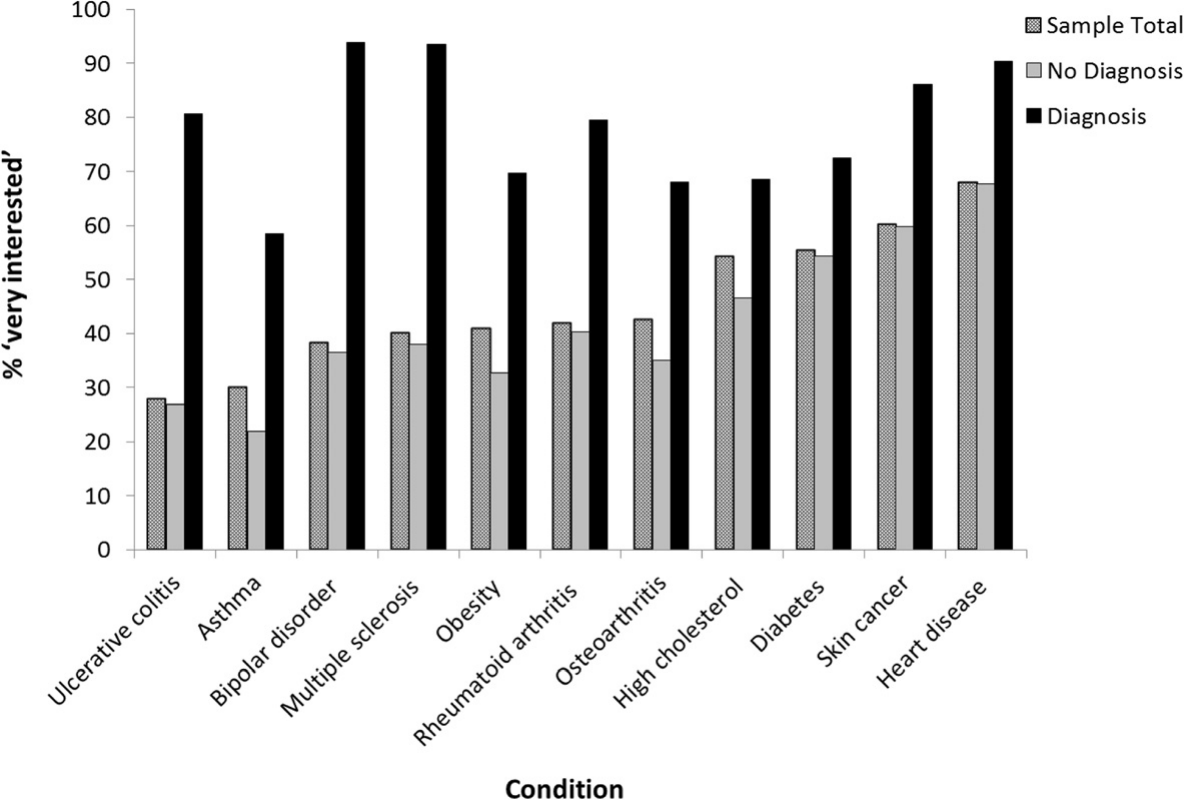
Proportion of individuals ‘very interested’ in genetic testing for a condition they already have versus those without a diagnosis of the condition

Of the 1,648 participants who completed the BL survey prior to viewing their results, 110 (6.7 %) were excluded due to missing data necessary for our analyses. Demographic characteristics of the remaining 1,538 participants included in our analyses are shown in Table 1. Compared to participants recruited from 23andMe, participants from Pathway were more likely to be women (67.3 % vs. 57.3 %, *P* <0.001); to have a positive screen for anxiety at baseline (13.8 % vs. 8.8 %, *P* = 0.002); to report lower levels of education (global *F*-test *P* <0.001) and lower self-reported health (global *F*-test *P* <0.001); and tended to be younger (mean age = 44.3 vs. 51.2, *P* <0.001) (Additional file 1: Table S1). The proportion of participants who reported that they were ‘very interested’ in PGT results for each of the 11 conditions ranged from 28.0 % (ulcerative colitis) to 68.1 % (heart disease) (Fig. 1). A majority of participants were very interested in results for high cholesterol, type 2 diabetes, skin cancer, and heart disease. Only 227 participants (14.8 %) were very interested in PGT for all 11 conditions, and even fewer (n = 29, 1.9 %) stated that they were not at all interested in PGT for any of the 11 conditions. Figure 1 shows the proportion of participants who were very interested in PGT results for each condition, stratified by personal diagnosis status. Across all conditions, participants who reported a personal diagnosis of a given condition were more likely to be very interested in PGT results for that condition than participants who did not report it. Condition-specific interest was consistently higher among participants from Pathway, but no clear pattern of differences in frequency of personal diagnosis and family history was noted across companies (Additional file 1: Table S2).

The proportion of participants who reported a personal diagnosis of each of the 11 conditions ranged from 2.0 % (heart disease, ulcerative colitis) to 36.2 % (high cholesterol) (Table 2). Only five conditions (asthma, obesity, diabetes, osteoarthritis, high cholesterol) had a frequency greater than 15 %. The frequency of reported family history of each condition was typically higher, ranging from 6.9 % (multiple sclerosis) to 68.6 % (high cholesterol) (Table 2).

**Table 2:** Individuals with personal diagnosis and family history of each condition, and logistic regression examining the association with interest among customers

Condition	n (%)	OR^a^	95 % CI	*P* value
Ulcerative colitis				
No diagnosis/No FH		1		
Diagnosis	31 (2.0)	6.83	2.21-21.12	0.001
FHx	93 (9.0)	2.54	1.59-4.30	<0.001
Asthma				
No diagnosis/No FH		1		
Diagnosis	341 (22.2)	2.86	1.66-4.90	<0.001
FHx	552 (36.5)	1.82	1.43-2.31	<0.001
Bipolar disorder				
No diagnosis/No FH		1		
Diagnosis	48 (3.1)	13.54	1.74-105.15	0.013
FHx	226 (19.9)	3.23	2.29-4.54	<0.001
Multiple sclerosis				
No diagnosis/No FH		1		
Diagnosis	62 (4.0)	19.99	4.57-87.35	<0.001
FHx	76 (6.9)	4.14	2.40-7.00	<0.001
Obesity				
No diagnosis/No FH		1		
Diagnosis	350 (22.8)	2.35	1.17-5.4.71	0.016
FHx	750 (49.4)	1.70	1.36-2.12	<0.001
Rheumatoid arthritis				
No diagnosis/No FH		1		
Diagnosis	63 (4.1)	3.87	1.97-7.60	<0.001
FHx	199 (15.0)	1.55	1.21-1.96	0.005
Osteoarthritis				
No diagnosis/No FH		1		
Diagnosis	353 (23.0)	2.51	1.63-3.87	<0.001
FHx	593 (44.7)	1.49	1.17-1.90	0.001
High cholesterol				
No diagnosis/ No FH		1		
Diagnosis	556 (36.2)	2.98	1.86-4.76	<0.001
FHx	1039 (68.6)	1.64	1.31-2.06	<0.001
Diabetes				
No diagnosis/No FH		1		
Diagnosis	94 (6.1)	2.07	1.28-3.37	0.003
FHx	604 (39.3)	2.43	1.97-2.99	<0.001
Skin cancer				
No diagnosis/ No FH		1		
Diagnosis	36 (2.3)	4.49	1.47-13.69	0.008
FHx	282 (18.3)	2.36	1.74-3.20	<0.001
Heart disease (coronary artery)				
No diagnosis/No FH		1		
Diagnosis	31 (2.0)	3.99	1.18-2.17	0.018
FHx	575 (56.7)	2.10	1.66-2.62	<0.001

^a^The model for each condition includes history of personal diagnosis and family history of that condition, and is adjusted for age, sex, race, ethnicity, education, company from which the test was purchased along with self-reported health and diagnosis of any other medical condition

Results of the logistic regression analyses are presented in Table 2. After adjustment for age, gender, race, ethnicity, education, anxiety, PGT company, and diagnosis of any other condition, both personal and family history of a condition were significantly associated in all cases with interest in PGT results for that condition. Odds ratios for the effect of a personal history on interest ranged from 2.07 (diabetes) to 19.99 (multiple sclerosis), while odds ratios for the effect of a family history on interest were in most cases smaller, ranging from 1.55 (rheumatoid arthritis) to 4.14 (multiple sclerosis).

## Discussion

Using baseline data from the PGen Study, we demonstrated that consumer interest in genetic risk information from PGT is independently associated both with having a family history of the condition and with having the condition itself. This finding is surprising because the emphasis of PGT marketing and discussion is typically focused on predicting future health risks. Although earlier work has often focused on the role of family history of a disease as motivation to obtain PGT [11, 12], the notion that DTC customers could be most interested in information on the etiology of their own disease condition has not yet been raised in discussions about the utility of PGT.

Comparisons of odds ratios across conditions encourage the speculation that rarer conditions, and conditions without firmly established etiology (such as multiple sclerosis, bipolar disorder, and ulcerative colitis), could evoke more interest in a genetic explanation than more common conditions (obesity, elevated cholesterol, and heart disease). Thus, it is possible that individuals who decide to purchase PGT anticipate deriving psychological benefit from information that might appear to shed light on the etiology of a disease they already have. Even for a condition that is as common as obesity, and where strong public beliefs about etiology exist, overweight participants in our prior qualitative studies described relief of guilt and self-blame after learning about their increased genetic risk of obesity, without any negative impact on motivation to ‘battle their biology’ [13, 14]. These findings match those from the area of genetic testing for mental health conditions, which also show that awareness of genetic risk may help to relieve rumination about causes of mental illness [24]. Alternatively, it is possible that individuals want to receive information on a genetic contribution to their personal illness in order to share this information with children or other family members for the purposes of risk evaluation and disease prevention [11, 12]. A third possibility is that customers believe knowledge about the underlying genetics of a condition could improve treatment outcomes, perhaps through pharmacogenomics. Since this is the first large-scale study to suggest an explanatory value of PGT for an existing condition, the findings need to be replicated and it will be important to carry out further qualitative and quantitative research to explore the reasons behind this phenomenon.

Our findings are also consistent with several previous studies that have demonstrated that genetic testing for a particular condition is motivated by family history of that condition [10, 12]. For example, a systematic review of 23 studies investigating uptake for breast cancer predictive genetic testing, either through actual uptake or anticipated uptake in hypothetical scenarios, found that individuals with a family history of cancer were twice as likely to have genetic testing for cancer risk as those without a family history [25]. Likewise, in a population-based survey, family history of heart disease was highly predictive of interest in genetic testing for heart disease [26].

This analysis had several strengths. The extensive information on demographics and family history collected in the PGen Study allowed us to control for possible confounding by the factors most commonly cited as having an impact on interest in genetic testing. The number of participants with each diagnosis was high enough to conduct the analysis, and we observed the effect regardless of condition, which increased our confidence in the validity of the findings. However, the study also had important limitations. Due to the small numbers of participants with a prior diagnosis for some of the diseases, confidence intervals were very wide for some conditions. Further research is needed to clarify whether the effect will also be observed in samples where a greater proportion has been diagnosed. Interest in PGT for specific conditions was only assessed with a simple three-point ordinal scale, without considering follow-up questions about its motivations or origins of interest. Future research could include questions that examine this topic in greater detail; for example, by using open-ended questions. For the current analyses, we only looked at ‘ever’ diagnosis of a condition, not whether customers were currently suffering with this condition. This may explain why a large proportion of participants reported both ‘excellent’ health, and having a medical diagnosis. Since a subset of PGen Study participants were drawn from a health-based social networking site, it is likely that these participants were particularly interested in exploring their health condition in more depth than the average person. Because participation was voluntary and part of a research study, our participants may differ from the typical PGT customer, and will certainly differ from the general population. Finally, all of our data were collected using self-report, which has been shown to vary in accuracy [27] and may introduce an element of bias.

## Conclusions

In conclusion, data from the PGen Study showed that a prior diagnosis of a disease is significantly associated with interest in PGT for that condition, even after adjusting for variables commonly associated with increased interest in genetic testing; including family history. This correlation with interest in genetic testing has not been previously described in research on the benefits and harms of PGT, and it may be an important factor in evaluating the clinical and personal utility of personal genomic testing.

## Additional file

Additional file 1:
**Tables S1 and S2 contain descriptive statistics stratified by personal genomic testing company.** (DOCX 19 kb)
