# Supplementary material for: Explaining, not just predicting, drives interest in personal genomics
Source: Genome Med. 2015 Aug 1;7(1):74. doi: 10.1186/s13073-015-0188-5 (PMC4533947; doi:10.1186/s13073-015-0188-5)
Supplement: Additional file 1: — Tables S1 and S2 contain descriptive statistics stratified by personal genomic testing company. (DOCX 19 kb) [file 13073_2015_188_MOESM1_ESM.docx]

| **Supplemental Table 1**. Baseline demographic characteristics of PGen Study participants, stratified by personal genomic testing company | | | |
| --- | --- | --- | --- |
|  | **23andMe**  **(n = 976)** | **Pathway Genomics (n = 560)** | **X^2^ test**  **p-value** |
| **Variable** | n (%) | |  |
| Female | 559  (57.3) | 377  (67.3) | <0.001 |
| Non-White Race | 105  (10.7) | 49  (8.8) | 0.212 |
| Hispanic/Latino Ethnicity | 52  (5.3) | 32  (5.7) | 0.741 |
| Positive GAD-2 Screen for Anxiety/Panic Disorder | 86  (8.8) | 77  (13.8) | 0.002 |
| Highest Level of Education |  |  |  |
| < College Degree | 190  (19.4) | 150  (26.8) | <0.001 |
| College Degree | 282  (28.8) | 190  (33.9) |  |
| Some Graduate School | 365  (36.4) | 175  (31.3) |  |
| Doctoral Degree | 150  (15.3) | 45  (8.0) |  |
| Self-Reported Health |  |  |  |
| Poor | 14  (1.4) | 50  (8.9) | <0.001 |
| Fair | 77  (7.9) | 95  (17.0) |  |
| Good | 289  (29.6) | 176  (31.4) |  |
| Very Good | 416  (42.6) | 188  (33.6) |  |
| Excellent | 180  (18.4) | 9.1  (51) |  |
| Age, mean ± standard deviation | 51.24  ±15.88 | 44.32  ±13.74 | <0.001 |
| Abbreviations: GAD-2, Generalized Anxiety Disorder – 2 Item Scale; SD, standard deviation | | | |

| **Supplemental Table 2**. Disease-specific interest, diagnosis status, and family history among PGen Study participants, stratified by personal genomic testing company | | | |
| --- | --- | --- | --- |
|  | **23andMe**  **(n = 976)** | **Pathway Genomics**  **(n = 560)** | **X^2^ test**  **p-value** |
| **Variable** | n (%) | |  |
| **Ulcerative colitis**  High Interest  Diagnosis  Family History | 243 (24.9)  21 (2.1)  47 (7.0) | 187 (33.4)  10 (1.8)  46 (12.8) | <0.001  0.627  0.002 |
| **Asthma**  High Interest  Diagnosis  Family History | 265 (27.1)  199 (20.3)  327 (34.2) | 197 (35.2)  142 (25.4)  225 (40.3) | 0.001  0.022  0.018 |
| **Bipolar disorder**  High Interest  Diagnosis  Family History | 317 (32.5)  21 (2.1)  116 (16.0) | 272 (48.6)  27 (4.8)  110 (26.8) | <0.001  0.004  <0.001 |
| **Multiple sclerosis**  High Interest  Diagnosis  Family History | 314 (32.1)  1 (0.1)  40 (5.8) | 303 (54.1)  61 (10.9)  36 (8.9) | <0.001  <0.001  0.052 |
| **Obesity**  High Interest  Diagnosis  Family History | 367 (37.6)  216 (22.1)  464 (48.3) | 265 (47.3)  134 (24.0)  286 (51.3) | <0.001  0.396  0.250 |
| **Rheumatoid Arthritis**  High Interest  Diagnosis  Family History | 360 (36.8)  29 (3.0)  134 (15.8) | 285 (50.9)  34 (6.1)  65 (13.6) | <0.001  0.003  0.161 |
| **Osteoarthritis**  High Interest  Diagnosis  Family History | 375 (38.4)  224 (22.9)  357 (42.0) | 279 (49.8)  129 (23.1)  236 (49.4) | <0.001  0.950  0.010 |
| **High cholesterol**  High Interest  Diagnosis  Family History | 508 (52.0)  378 (38.7)  639 (66.8) | 330 (58.9)  178 (31.8)  400 (71.7) | 0.009  0.008  0.047 |
| **Diabetes**  High Interest  Diagnosis  Family History | 511 (52.3)  67 (4.8)  382 (46.8) | 344 (61.4)  27 (6.9)  222 (48.1) | 0.001  0.112  0.656 |
| **Skin cancer**  High Interest  Diagnosis  Family History | 546 (55.9)  28 (2.9)  148 (15.1) | 381 (68.2)  8 (1.4)  134 (23.9) | <0.001  0.073  <0.001 |
| **Heart disease (Coronary Artery Disease)**  High Interest  Diagnosis  Family History | 637 (65.2)  24 (2.5)  370 (56.1) | 409 (73.0)  7 (1.3)  205 (57.7) | 0.002  0.107  0.605 |
|  | | | |
